# Decoding the ZmNF-YC1–ZmAPRG pathway for phosphorus efficiency

**DOI:** 10.3389/fpls.2025.1548962

**Published:** 2025-03-19

**Authors:** Hafiz Athar Hussain, Saleem Uddin, Daofeng Liu, Wenjing Long

**Affiliations:** ^1^ College of Horticulture and Landscape Architecture, Southwest University, Chongqing, China; ^2^ Rice and Sorghum Institute, Sichuan Academy of Agricultural Sciences, National Sorghum Improvement Center Sichuan Branch, Deyang, Sichuan, China

**Keywords:** phosphorus deficiency, ZmNF-YC1-ZmAPRG module, NF-Y complex, lipid metabolism, photosynthetic efficiency

## Introduction

Phosphorus (P) deficiency is a significant limiting factor for cereal yields, particularly in maize (Dhillon et al., 2017). While the Nuclear Factor Y, C subunit (NF-YC) pathway has been extensively studied in the context of abiotic stresses such as drought, salinity, and temperature (Sato et al., 2014; Wu et al., 2018; Zheng et al., 2021), its role beyond P deficiency remains largely underexplored. Recent research highlights the ZmNF-YC1–ZmAPRG pathway as a promising mechanism for improving maize tolerance to low P (Bai et al., 2024). In this pathway, ZmNF-YC1 acts as a transcriptional activator, interacting with ZmNF-YB14 and ZmNF-YA4/10 to modulate acid phosphatase-regulating gene *(ZmAPRG)* for P homeostasis, lipid composition, and photosynthesis, contributing to plant resistance against P-deficit conditions. Notably, *ZmAPRG* is an uncharacterized gene identified through map-based cloning, which enhances acid phosphatase activity and phosphate concentration in maize leaves during phosphate starvation (Yu et al., 2019). However, P deficiency can severely impair plant growth and crop productivity, underscoring the importance of efficient P acquisition and utilization for agricultural sustainability (Paz-Ares et al., 2022; Zhu et al., 2022). A key aspect of this adaptive response is the regulation of P-responsive genes by various transcription factors (TFs), which orchestrate metabolic and physiological responses to P deficiency (Liu et al., 2024).

Generally, the PHT family of phosphate transporters remains the primary focus for improving phosphorus use efficiency (PUE) in maize due to their critical role in facilitating the uptake, translocation, and remobilization of inorganic phosphate (Pi) from the soil into plant roots (Nagy et al., 2006; Wang et al., 2017). Specifically, the PHT1 family members function predominantly under low P conditions and have been widely used to enhance PUE in plants. However, it was noted that, overexpression of OsPHR2 caused excessive Pi accumulation in shoots, which retarded whole-plant growth under sufficient Pi condition (Zhou et al., 2008). This excessive Pi accumulation due to PHR2 was attributed to the upregulation of phosphate transporters (PTs) in shoots, ultimately resulting in Pi toxicity (Li et al., 2014; Liu et al., 2010). So, these studies showed that PHR2 could cause Pi toxicity due to excessive accumulation in plants. Furthermore, Pi toxicity in rice has been associated with reduced Rubisco activation and inhibited photosynthesis, leading to lipid peroxidation (Takagi et al., 2020).

In contrast, the NF-YC transcription factors offer distinct advantages for improving PUE by regulating a broader range of genetic and physiological responses (Bai et al., 2024). Unlike PHT1 transporters, which are directly involved in Pi uptake, NF-YC TFs play a central role in orchestrating the expression of multiple downstream genes, including P transporters and genes involved in stress responses, metabolic pathways, and developmental processes. This regulatory versatility enables NF-YC TFs to integrate external and internal signals, ensuring more efficient adaptation to fluctuating environmental conditions and maintaining optimal PUE in maize (Zhang et al., 2023).

Previously, it was indicated that overexpression (OE) of TaNFYA-B1, a low P-inducible TF on chromosome 6B, significantly enhanced P uptake and grain yield in wheat under varying P supply conditions (Qu et al., 2015). However, OE of *ZmAPRG* was shown to enhance inorganic phosphate concentration and acid phosphatase activity, resulting in greater biomass in maize seedlings under low P conditions (Yu et al., 2019). Recently, Bai et al. (2024) reported that ZmNF-YC1, an upstream regulator of *ZmAPRG*, has emerged as a key modulator in maize (Zea mays L.) under low P conditions.

As part of the NF-Y complex, ZmNF-YC1 plays a central role in the ZmNF-YC1–ZmAPRG pathway, a crucial regulatory axis governing P metabolism and tolerance mechanisms. ZmNF-YC1 serves as the C subunit of the NF-Y complex, which forms a heterotrimer with NF-YA and NF-YB subunits (Bai et al., 2024). The NF-Y complex has been reported to binds the CCAAT box in target gene promoters, a well-characterized cis-regulatory element, to regulate gene expression under various stress conditions (Myers and Holt, 2018; Qu et al., 2015). However, in the case of ZmNF-YC1 binding to the promoter of *ZmAPRG*, Bai et al. (2024) did not specify whether the binding site was a CCAAT box or another motif. Thus, it remains unclear whether ZmNF-YC1 binds to the CCAAT box or a distinct phosphate-specific regulatory element. Future studies, such as mutagenesis of potential binding sites or ChIP-seq, are necessary to identify the precise binding site and confirm the regulatory mechanism.

## Regulatory mechanisms of the ZmNF-YC1–ZmAPRG pathway

**Figure 1:**
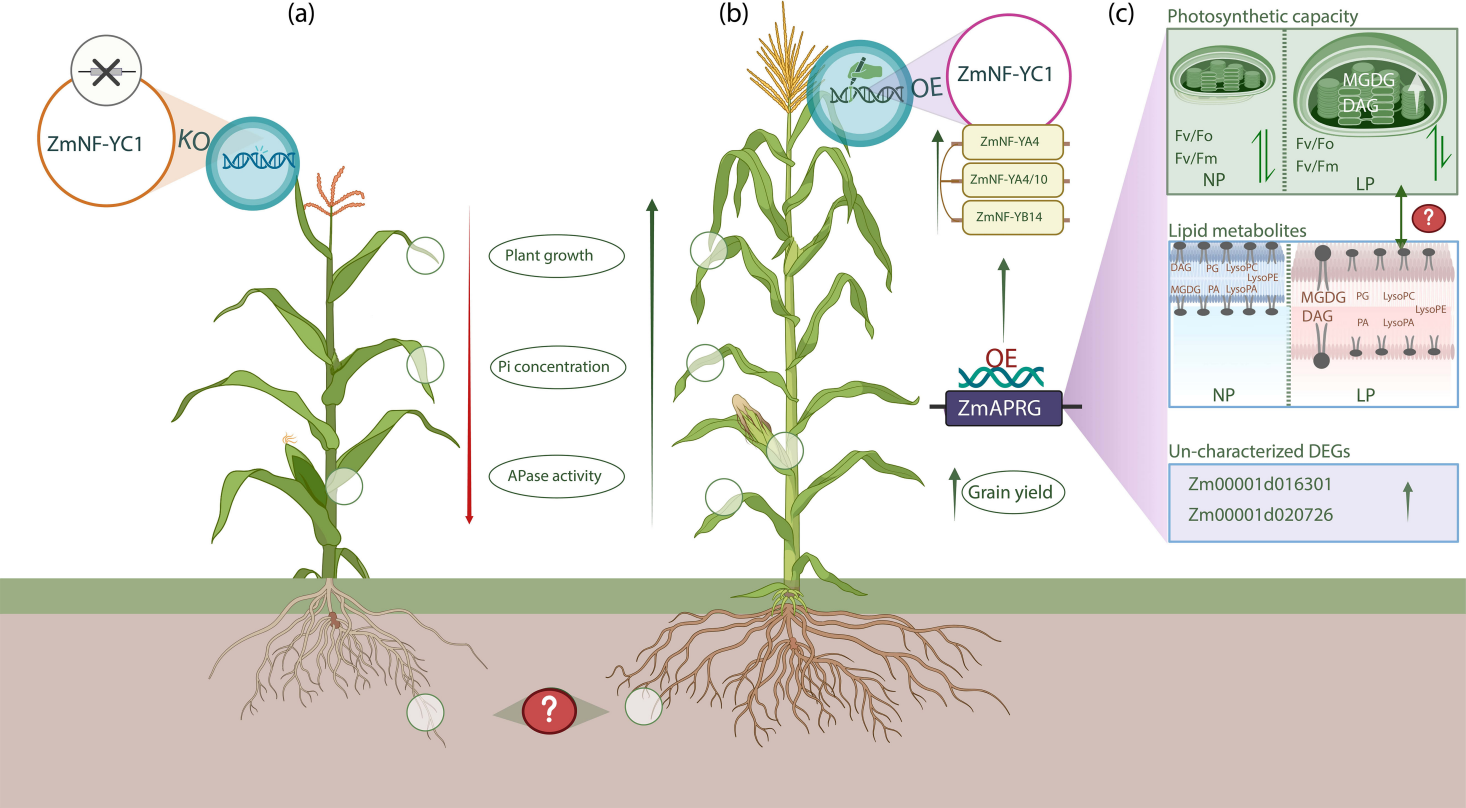
Role of ZmNF-YC1–ZmAPRG pathway in modulating maize tolerance to low P conditions. In part **(a)**, the ZmNF-YC1 knockout (KO) plant shows reduced plant growth, acid phosphatase (APase) activity and inorganic P (Pi) concentration. **(b)** illustrates the ZmNF-YC1 OE in maize plant, where ZmNF-YC1 forms complexes with ZmNF-YB14, ZmNF-YA4 and ZmNF-YA410 to activate the promoter of *ZmAPRG* (Bai et al., 2024). OE of *ZmAPRG* regulates photosynthetic efficiency and lipid metabolites, ultimately improve growth and grain yield of plants. In ZmNF-YC1–ZmAPRG pathway, significant effects were observed on above-ground traits, such as length and dry weight of shoot, while no effects were reported on root traits, including root length, biomass, and root system architecture. This suggests that the pathway primarily influences shoot growth under low P conditions, leaving the impact on root development unclear. Since P deficiency typically induces root adaptations for enhanced nutrient acquisition, the absence of root effects raises question. Could compensatory mechanisms or alternative pathways govern root adaptations for nutrient acquisition in ZmNF-YC1–ZmAPRG OE plants? How might further analysis of root molecular responses and architectural changes provide deeper insights into the role of this pathway in P tolerance? Part **(c)** illustrates the impact of *ZmAPRG* OE on lipid remodeling, photosynthetic capacity, and the regulation of lipid transport and metabolism-related genes. Two differentially expressed genes (DEGs) identified as phosphatase-related suggest that *ZmAPRG* not only regulates lipid metabolites and photosynthetic efficiency but may also influence phosphatase activities through novel pathways. Regarding photosynthetic capacity, there is no significant difference in chlorophyll fluorescence parameters; Fv/Fm and Fv/Fo values under NP conditions, but in LP conditions, *ZmAPRG*-OE plants exhibit significantly increased Fv/Fm and Fv/Fo values. Under normal P (NP) conditions, phospholipids such as LysoPE, LysoPC, LysoPA, PA and PG remain unchanged. However, in low P (LP) conditions with *ZmAPRG* OE, non-phosphorus lipids like MGDG and DAG increase, while phospholipids decrease (Bai et al., 2024). This induction of MGDG and DAG under LP conditions is likely a compensatory mechanism for phospholipid degradation, aimed at maintaining membrane stability. Membrane stability plays a critical role in supporting photosynthetic function, which could explain the observed improvement in photosynthetic efficiency. While, the improvement in photosynthetic efficiency correlates with changes in lipid metabolites, particularly the increase in MGDG and DAG (Boudière et al., 2014), it is unlikely that the elevated levels of MGDG and DAG directly enhance photosynthesis. Instead, their role may be limited to stabilizing the thylakoid membranes, which indirectly supports photosynthetic processes. Unraveling whether MGDG and DAG have a direct impact on photosynthesis remains a key question for future research.

The NF-YB and NF-YC subunits initially form a heterodimer, which subsequently recruits NF-YA proteins to assemble the complete NF-Y transcriptional complex, responsible for regulating the expression of downstream target genes (Zheng et al., 2021; Sato et al., 2014; Hou et al., 2014). In this process, NF-YC first interacts with NF-YB to form dimers in the cytoplasm, which then translocate to the nucleus where they associate with NF-YA to form the functional heterotrimeric complex that modulates gene expression (Zhang et al., 2023). Under low P availability, ZmNF-YC1 forms a functional heterotrimeric complex with ZmNF-YB14, ZmNF-YA4, and ZmNF-YA10. Initially, ZmNF-YC1 and ZmNF-YB14 form a heterodimer in the cytoplasm, which then recruits ZmNF-YA4 and ZmNF-YA410 to complete the complex in the nucleus. Notably, ZmNF-YB14 is found in both the cytoplasm and nucleus, while ZmNF-YA4 and ZmNF-YA10 are localized only in the nucleus (Bai et al., 2024). Once the ZmNF-YC1, ZmNF-YB14, ZmNF-YA4 and ZmNF-YA10 complex is assembled, it binds to the promoter of *ZmAPRG*. This binding enhances the transcription of *ZmAPRG*, which regulates lipid composition adjustment, and photosynthetic capacity. Under low P conditions, *ZmAPRG* influences the expression of lipid transport and metabolism-related genes (Bai et al., 2024), including Zm00001d047447, which encodes phospholipase C involved in phospholipid hydrolysis (Nakamura et al., 2005), and Zm00001d044136, which encodes the GTAP protein that catalyzes glycerol biosynthesis and supports membrane stability and stress response (Xue et al., 2019). In response to P deficiency, phospholipids are hydrolyzed to release P, and non-phosphorus lipids like MGDG and DAG replace them to maintain membrane stability (Zhu et al., 2022). *ZmAPRG* OE increases MGDG and DAG while reducing phospholipids, which are key components of photosynthetic membranes. Although *ZmAPRG* improves photosynthetic capacity under low P, whether the increase in MGDG and DAG directly boosts photosynthesis remains unclear and requires further investigation (**Figure 1**).

## Expanding beyond phosphorus: is the ZmNF-YC1–ZmAPRG pathway key to resilience against multiple stresses?

Plants frequently encounter multiple simultaneous stresses in their natural environment, and increasing evidence suggests that TFs involved in one stress response pathway can cross-communicate with other pathways (Shaik and Ramakrishna, 2014). A key example is NF-YC TFs, which is activated in response to low P conditions (Bai et al., 2024), and drought or salinity stresses (Chen et al., 2015). NF-YC plays a pivotal role in modulating plant stress responses as monomers, complexes, or in coordination with other TFs (Zhang et al., 2023).

Previous studies have shown that NF-Y complexes are key regulators of stress resilience in response to drought, salinity and temperature stresses (Wu et al., 2018; Zheng et al., 2021; Sato et al., 2014, 2019). For instance, GmNF-YC14 forms a heterotrimer with GmNF-YA16 and GmNF-YB2 to activate the GmPYR1-mediated ABA signaling pathway, regulating drought response in soybean, as confirmed by gene knockout and overexpression studies (Yu et al., 2021). Likewise, overexpression of NF-YB2 and NF-YB3 enhanced drought and heat stress tolerance in Arabidopsis, while knockout mutants exhibited increased sensitivity to these stresses (Sato et al., 2019). Beyond stress adaptation, NF-Y TFs also regulate nutrient homeostasis. In wheat, TaNFYC genes play a significant role in adapting to nitrogen (N) and P deficiency, promoting nutrient homeostasis (Qu et al., 2015). In Arabidopsis, the interaction between QQS protein and NF-YC4 shows that NF-Y regulates carbon and nitrogen allocation, balancing protein and starch levels for developmental homeostasis (Li et al., 2015). In *Brassica napus*, NF-YC genes have shown distinct tissue-specific roles in response to nitrogen deficiency (Zheng et al., 2021). These findings suggesting that the ZmNF-YC1 complex might also play a role in modulating pathways that respond to multiple stress conditions.

While ZmNF-YC1 is established as crucial in P deficiency response, its potential role in integrating multiple stress signals remains unclear. Specifically, although it is hypothesized that ZmNF-YC1–ZmAPRG pathway could influence root architecture and potentially improve water uptake efficiency, thus enhancing drought resilience and PUE under stress conditions, this relationship remains speculative. Given its regulatory versatility, ZmNF-YC1 may extend beyond P homeostasis, orchestrating crosstalk between multiple nutrient pathways and contributing to overall stress adaptation.

The mechanisms underlying water utilization and P absorption are distinct and complex, and there is currently no direct experimental evidence linking *ZmAPRG* to enhanced water uptake capabilities. In maize, the ZmNF-YC1 complex, which adapts P deficiency through a heterotrimeric complex formation with ZmNF-YB14, ZmNF-YA4 and ZmNF-YA410 (Bai et al., 2024), might extend its regulatory functions to other stress-responsive genes. One critical outcome of the ZmNF-YC1–ZmAPRG pathway is its regulation of lipid metabolism under low P conditions, which plays a key role in maintaining membrane integrity under osmotic stresses such as drought and salinity. The lipid remodeling that occurs under low P conditions, which shifts from phospholipid-based to non-phospholipid-based lipids, could similarly support membrane stability in high-salinity environments, preventing excessive ion leakage and preserving cellular function. This positions ZmNF-YC1 as a valuable target for breeding multi-stress-tolerant maize varieties capable of thriving in diverse environmental conditions, offering a significant contribution to sustainable agriculture amid rapid climate change.

## Challenges and future perspectives

ZmNF-YC1 is a transcriptional regulator under low P conditions, forming complexes that activate *ZmAPRG*, which modulates gene expression of lipid transport and metabolism, photosynthetic capacity, improve resistance against P deficiency. However, the identification of the ZmNF-YC1–ZmAPRG pathway as a key mechanism in P deficiency response is groundbreaking (Bai et al., 2024). However, role of ZmNF-YC1–ZmAPRG pathway on root architecture, such as enhanced root hair growth, root biomass and lateral root formation, under low P conditions is unclear, which need to investigate (**Figure 1**). Under low P conditions, *ZmAPRG* OE increases the levels of non-phospholipid MGDG and DAG, and reduce the levels of phospholipids (Bai et al., 2024), which are key components of photosynthetic membranes (Boudière et al., 2014). The enhanced photosynthetic capacity observed with *ZmAPRG* OE suggests that *ZmAPRG* plays a key role in low P tolerance by regulating photosynthesis. However, while increased MGDG and DAG levels correlate with improved photosynthesis under low P, the direct relationship between these lipid changes and photosynthetic improvement requires further investigation. Given its influence on P metabolism, lipid composition, and photosynthesis, ZmNF-YC1 could be a pivotal factor in improving both nutrient-use efficiency and water-use efficiency in maize. Moreover, understanding this pathway’s interaction with other stress responses, modifying these genes could enhance their resilience to both P deficiency and other abiotic stresses, such as heat, salinity and drought. If ZmNF-YC1 contributes to managing multiple stress responses, it could become a pivotal target for breeding maize varieties with enhanced resilience to a range of environmental challenges.

Furthermore, the potential application of ZmNF-YC1–ZmAPRG pathway in other crops should be tested in low P conditions. If homologs of NF-YC and APRG exist in other cereals like rice or wheat, manipulating these genes could enhance P use efficiency across a range of agricultural systems, potentially reducing reliance on phosphate fertilizers. Further research should focus on identifying other co-regulatory factors and downstream target genes involved in the ZmNF-YC1–ZmAPRG pathway to provide a more comprehensive understanding of its regulatory network. Large-scale field trials are also necessary to confirm the effectiveness of this pathway across diverse maize genotypes and environmental conditions, ensuring its applicability in real-world agricultural systems.

ZmNF-YC1–ZmAPRG pathway could be a target for precision breeding, which could involve enhancing the expression of *ZmAPRG* or similar genes in other crop species, leading to more efficient nutrient use and greater yields under low P conditions. Moreover, ZmNF-YC1 and *ZmAPRG* are key target genes that could be directly utilized in breeding programs or modified through gene-editing technologies to enhance P-use efficiency under low P conditions.
